# Development of a Social Media Campaign to Support HIV Prevention and Care Among Transgender Latina Women: Community-Engaged Mixed Methods Feasibility Pilot Study

**DOI:** 10.2196/79606

**Published:** 2026-01-27

**Authors:** Jane J Lee, Kathleen Agudelo Paipilla, Joel Aguirre, Patricia Alarcon, Yesenia Cruz, Martha Zuniga, Juliann Li Verdugo, Katie Vo, E Roberto Orellana, Susan M Graham

**Affiliations:** 1 Suzanne Dworak-Peck School of Social Work, University of Southern California, 669 W 34th Street, Los Angeles, CA, 90089, United States, 1 213-821-9657; 2 School of Social Work, University of Washington, Seattle, WA, United States; 3 Department of Global Health, University of Washington, Seattle, WA, United States; 4 Entre Hermanos, Seattle, WA, United States; 5 School of Public Health, University of Washington, Seattle, WA, United States; 6 School of Medicine, University of Washington, Seattle, WA, United States

**Keywords:** community engagement, digital health, eHealth, health disparities, HIV prevention, latina, social media, transgender

## Abstract

**Background:**

Transgender Latina women in the United States face disproportionate HIV risk due to intersecting social and structural vulnerabilities that limit access to care. While gender-affirming, culturally responsive, and eHealth strategies show promise for improving access, social media–based approaches remain underused despite their potential to reach marginalized groups at scale.

**Objective:**

This study aimed to develop and pilot a culturally tailored social media campaign to increase awareness of HIV prevention and care services offered by a community-based organization (CBO) in King County, Washington, for transgender Latina women and to assess the campaign’s feasibility and acceptability.

**Methods:**

We conducted a community-engaged, mixed methods pilot study using a multiphase design. In phase 1, we conducted cross-sectional, in-depth interviews with transgender Latina women (n=20) recruited by a CBO in King County, Washington. Interviews were analyzed using thematic analysis, guided by the Unified Theory of Behavior, to inform campaign messaging priorities. A subsequent focus group (n=7) then reviewed and refined 6 draft campaign concepts according to the community preferences. In phase 2, the finalized campaign was piloted on Facebook and Instagram. A cross-sectional REDCap (Research Electronic Data Capture; Vanderbilt University) survey was conducted with a subset (n=100) of transgender Latina women exposed to the campaign who voluntarily consented to complete the survey after being directed from the campaign. Survey data were summarized using descriptive statistics to assess campaign reach and feasibility and acceptability outcomes.

**Results:**

In-depth interview participants were a mean age of 37.6 (SD 9.5) years and reported an average of 10.2 (SD 10.8) years residing in the United States (n=20). Interviews revealed four key themes: (1) importance of HIV prevention and awareness, (2) accessibility of HIV services, (3) provision of culturally tailored care, and (4) need for confidentiality. Among survey respondents (mean age of 29.7, SD 5.2 years), 97% (97/100; 95% CI 91.5%‐99.0%) had ever tested for HIV and 44% (44/100; 95% CI 34.3%‐53.7%) reported testing within the past 6 months. A total of 3 respondents were living with HIV, all on antiretroviral therapy. Nearly all (91/100, 91%; 95% CI 84.3%‐95.2%) reported campaign-motivated action, including HIV testing or seeking information or services.

**Conclusions:**

Findings demonstrate the feasibility and acceptability of a culturally tailored campaign, cocreated with community members, to promote HIV prevention and care among transgender Latina women. By integrating participatory methods with digital outreach, this study contributes an innovative model that centers community voices in campaign design while leveraging widely used platforms. The study has implications for providing CBOs with scalable, low-cost strategies to expand culturally responsive HIV services, reduce stigma, and motivate health-seeking behaviors in populations often overlooked by mainstream public health messaging. This work underscores how codesigned social media campaigns can complement traditional outreach and inform future HIV prevention strategies for underserved populations.

## Introduction

Transgender women, particularly transgender women of color, experience heightened risk of HIV transmission in the United States [1]. Most recent data from the National HIV Behavioral Surveillance Among Transgender Women, which monitors behavioral risk factors, HIV testing behaviors, and receipt of prevention services among transgender women in 7 US urban areas, reported that 42% of the 1608 transgender women interviewed between 2019 and 2020 were living with HIV [2]. Hispanic or Latina transgender women are disproportionately affected, accounting for over one-third of all new HIV diagnoses among transgender women [2,3].

Transgender Latina women experience multiple and intersecting forms of marginalization including stigma, discrimination, and violence related to both their gender and ethnicity [4]. Many are immigrants who may face additional challenges, including lack of insurance, unemployment, homelessness, and poor mental health [5,6]. These social and structural vulnerabilities increase their risk of HIV transmission and impede engagement in health services [7,8]. Moreover, transgender Latina women may have limited social support in the United States, which may further isolate them from community resources and exacerbate barriers to care [9]. Social isolation can undermine trust in health care systems and reduce awareness of available HIV prevention and treatment services, which can lead to delays in HIV testing and care [10,11]. These barriers contribute to low uptake of effective HIV prevention strategies such as pre-exposure prophylaxis (PrEP) in this population [12].

There is growing recognition that gender-affirming, person-centered services that address both social and cultural barriers are essential to improving engagement in HIV prevention and care for transgender populations [13,14]. However, there remains a dearth of HIV prevention intervention approaches that have been designed specifically for transgender Latina women and their specific needs [15]. While community-based service approaches have demonstrated effectiveness in improving service uptake [13], ensuring awareness and accessibility of these services within the transgender Latina community, again, remains a significant challenge. Specifically, approaches to reach this population must be culturally grounded and responsive, acknowledging and addressing the unique cultural, social, and structural contexts that shape the lives and identities of transgender Latina women [16].

Notably, mobile apps and eHealth offer opportunities to engage marginalized groups disconnected from traditional health care and deliver information that addresses structural barriers [17-19]. Social media platforms, which are widely used within Latino communities, remain an underused tool for expanding HIV prevention efforts to vulnerable populations [18,20]. In prior work, we developed and tested a social media campaign promoting HIV prevention among Latino gay, bisexual, and other men who have sex with men, which successfully reached the target population [21]. However, transgender Latina women face distinct social, cultural, and structural barriers that can differ from those of cisgender Latino men, including gender-based stigma. To reach transgender Latina women, therefore, required the development of a campaign to ensure that content reflected their lived experiences, preferences, and unique prevention needs. This called for qualitative inquiry to capture nuanced perspectives and community-driven insights to inform the design and framing of culturally relevant social media content [22].

We sought to develop a social media campaign for transgender Latina women to increase awareness of HIV prevention and care services offered at Entre Hermanos, a community-based organization (CBO) providing bilingual HIV testing and PrEP navigation support to Latino LGBTQ+ (lesbian, gay, bisexual, transgender/transsexual, queer, and other minority sexual orientations and gender identities) individuals in King County, Washington. The objectives of this paper are to describe the development process of the campaign and present pilot data on its preliminary feasibility and acceptability.

## Methods

### Research Design Overview

This study was conducted in the state of Washington from October 2023 to December 2024 and involved two phases: (1) the first phase involved qualitative interviews and a focus group with transgender Latina women to guide development of the social media content and campaign; and (2) the second phase involved piloting the campaign on social media platforms and assessing its feasibility via an online survey with transgender Latina women.

### Phase 1: Development of Social Media Content and Campaign

#### Methods

For the first phase, we used a 2-step qualitative approach to conduct in-depth interviews and a focus group with transgender women who identified as Hispanic or Latina. A qualitative approach was particularly suited to explore preferences, perceived barriers, and culturally relevant communication strategies that may not be easily captured through quantitative methods.

Interviews were conducted between November 2023 and May 2024 with 20 participants, a sample size determined to be sufficient for achieving information saturation for our objectives [23]. Following the interviews, a focus group was conducted with participants in June 2024 to obtain feedback on the social media content developed from the interview findings. Approximately 5 to 7 participants were sought as an ideal size to promote open discussion and ensure that each participant had the opportunity to share detailed feedback.

#### Study Participants

Eligibility criteria for study participation included (1) identifying as Hispanic or Latina or Latinx individuals, (2) age greater than or equal to 18 years, (3) identifying as transgender, (4) assigned male at birth, and (5) living in Washington.

#### Participant Recruitment

Participants were recruited through Entre Hermanos, a Latino-serving CBO in King County, Washington. We purposively recruited participants via multiple community outreach methods designed to reach transgender Latina women engaged with Entre Hermanos’ programs and broader community networks. Specifically, Entre Hermanos staff members who were part of the research team contacted potentially eligible participants to invite them to participate. In addition, recruitment materials were disseminated through the organization’s email listserv, which advertised the study and provided contact information for enrollment. Participants were purposively recruited to ensure diversity in age and length of time living in the United States, which were factors considered important in shaping social media engagement.

In-depth interviews and focus groups were conducted in person at Entre Hermanos in a private, confidential space. The location was selected collaboratively by the research team and Entre Hermanos staff, given participants’ trust and familiarity with the organization as a community-based setting.

#### Data Collection

##### In-Depth Interviews

All interested participants were screened for eligibility and consented to participate in the study. Prior to the interview, participants completed a brief demographic survey that asked about sociodemographic characteristics (eg, age, education, country of origin, current gender identity, assigned sex at birth, and time living in the United States).

In-depth interviews were semistructured and were guided by the Unified Theory of Behavior (UTB) [24] to explore factors related to normative pressures, beliefs and expectancies, self-concept or image, affect and emotions, and self-efficacy in relation to engaging in HIV services [25]. The UTB provided a comprehensive framework for understanding behavioral intentions and actions and incorporates multiple determinants, including individual and contextual level factors, that aligned closely with the goals of this formative phase. Interviews also asked about preferences and behaviors related to social media use to assess preferred platforms, content, and delivery strategies in relation to information about HIV and prevention for transgender Latina women. Interviews were conducted in person by 2 trained native Spanish speakers (KAP and JA) and lasted approximately 1 hour. Interviewers were bicultural and bilingual and had extensive experience working with Hispanic or Latino and transgender populations.

##### In-Depth Interview Analysis

Interviews were audio recorded and transcribed verbatim by 2 native Spanish speakers (PA and KAP). Data were analyzed in Spanish using thematic analysis, which led to the identification of main themes. Thematic analysis followed a primarily deductive approach guided by the UTB, which informed the interview guide and coding framework. However, inductive coding was also applied to capture emergent themes that extended beyond the predefined theoretical constructs. A total of 2 coders independently reviewed and open-coded the interview transcripts, repeatedly reading to become familiar with the data and produced detailed summaries with notes on key points and potential themes and subthemes. The coders then met to compare findings, resolve discrepancies, and refine the themes through iterative discussion [22,26]. The transcripts were coded using Microsoft Word. A final table of main themes was developed and reviewed with the study principal investigator to contextualize the findings within existing literature.

##### Focus Group

Following the analysis of the in-depth interviews, a communication agency was engaged to develop a creative concept proposal for the campaign. A focus group (n=7) was then conducted to obtain feedback and suggestions on the proposed social media content. Focus group questions were guided by five domains from the UTB influencing health behaviors [24]: (1) normative pressures and perceived norms, (2) beliefs about consequences of the behavior, (3) self-concept and perceived identity of individuals engaging in the behavior, (4) affective and emotional responses, and (5) self-efficacy and perceived ability to overcome barriers [27,28]. Additional feedback on design, messaging, graphics, and format preferences was also collected. The focus group was conducted in person by 2 trained native Spanish speakers with extensive experience working with the transgender Latina community and lasted approximately 2 hours.

##### Focus Group Analysis

Focus group data were analyzed using thematic analysis and a structured, collaborative approach. While sessions were recorded, detailed notes taken by 2 research team members served as the primary data source, with recordings used for verification. Each note taker independently summarized the discussions, identifying key points and emerging themes. The researchers then compared summaries to reconcile differences and reach consensus on key findings. This approach is consistent with a rapid qualitative analysis approach, which is an applied qualitative method designed to obtain actionable and targeted insights on a shorter timeline than traditional qualitative methods [29,30]. Given that the focus group was conducted to refine the social media content rather than to develop a new theory, this method was appropriate for the study’s aims. A table was created to systematically organize themes across expression, general idea, visuals, and description for each campaign component [30]. This table was reviewed with the study team to finalize themes and ensure accuracy before sharing with the communication agency to revise campaign content.

##### Positionalities and Potential Biases of the Research Team

The research team included individuals with diverse backgrounds and lived experiences, including members who identified as transgender, Latina, immigrant, and lesbian, gay, bisexual, or queer. Community members were integral parts of the research team and contributed to all stages of the study, including data collection procedures and interpretation to ensure that decisions were grounded in community priorities and perspectives. This collaborative structure fostered reflexivity and ongoing dialogue about potential biases and assumptions [31]. Throughout the process, the team remained attentive to positionality and power dynamics, intentionally centering community voices and lived experiences in the interpretation of findings.

### Phase 2: Feasibility of Social Media Campaign Through Online Surveys

#### Methods

Based on findings from phase 1, a social media campaign was developed and piloted on Facebook and Instagram between November 2024 and December 2024. The campaign included 4 video posts promoting HIV prevention and care at Entre Hermanos among transgender Latina women. Each social media post included text inviting interested viewers to participate in an online survey. Clicking on the post directed individuals to a secure REDCap (Research Electronic Data Capture; Vanderbilt University) link, where they first reviewed and electronically signed an informed consent form [32]. Respondents were then screened for eligibility before completing the full survey.

#### Inclusion and Exclusion

Eligibility criteria for survey participation included: (1) self-identifying as Hispanic or Latina; (2) self-identifying as transgender; (3) reporting assigned male sex at birth; and (4) being at least the age of 18 years. Participants who did not meet these criteria were not eligible to participate.

#### Measures

The survey collected data on sociodemographic characteristics, HIV prevention behaviors, and reactions to the campaign content developed from previously validated instruments. Sociodemographic variables included age, country of birth, immigration status, education, employment, income, health insurance status, and religion. Behavioral variables included alcohol and drug use, sexual behaviors, and HIV prevention and care behaviors (HIV status, HIV testing history, HIV testing intentions, antiretroviral therapy [ART] use, and PrEP use). These behavioral variables were included as exploratory measures to provide information about participants’ HIV-related risk and prevention behaviors. These behavioral patterns informed the interpretation of campaign feasibility and reach.

Participants were also asked to rate the relevance of the campaign content and indicate whether the campaign influenced them to take any HIV-related action. Surveys were offered in Spanish or English and capped at 100 completed responses. Eligible participants received a US $25 incentive for survey completion.

#### Data Analyses

Survey data were analyzed descriptively to assess the characteristics of transgender Latina women reached by the campaign and to assess whether the campaign could successfully engage this population. Descriptive analyses were used to summarize participant profiles and assess campaign feasibility and acceptability.

### Ethical Considerations

The study was reviewed by the University of Washington Institutional Review Board and deemed exempt from federal human subjects regulations (study ID: STUDY00018207). In-depth interview and focus group participants provided verbal informed consent in accordance with approved study procedures, while survey participants completed electronic informed consent through the REDCap platform. In-depth interview and focus group participants received a US $50 incentive, and survey participants received a US $25 incentive for completion. Incentive amounts were determined in collaboration with our community partner to ensure appropriateness for participants’ time and effort across study activities. All qualitative and quantitative data were deidentified for analysis. To ensure privacy and confidentiality, only study team members were present during data collection, and all participants provided informed consent emphasizing voluntary participation and data confidentiality. Participants were informed that they could decline to answer any question or withdraw from the study at any time. They were reminded that some questions might be sensitive or emotionally distressing. In such cases, the investigator was prepared to provide referrals to counseling services for additional support. No participants reported distress or required referral during data collection. Focus group participants contributed to the development of the social media campaign and are thus identifiable in MultimediaAppendices 1^4^. Consent has been granted from all identifiable individuals for inclusion of their images and videos in this paper.

## Results

### Phase 1: In-Depth Interviews and Focus Group

#### Overview

Table 1 presents the sociodemographic characteristics and HIV-related behaviors of the in-depth interview and focus group participants (n=26). There was no missing data across all variables. The average age of participants was 37.6 (SD 9.5) years and 13 out of 26 (50%) were born in Mexico. No participants reported being married, and 15 out of 26 (58%) participants reported having health insurance. The majority (24/26, 92%) had previously tested for HIV, and 15 out of 26 (58%) participants were currently using PrEP.

**Table 1.** Sociodemographic characteristics and HIV-related behaviors of transgender Latina women who participated in in-depth interviews and a focus group in the state of Washington (November 2023-June 2024; n=26).

Variables	Value^a^
Sociodemographic characteristics	
Age (y), mean (SD; range)	37.6 (9.5; 19.0‐54.0)
Time in the United States (y), mean (SD; range)	10.2 (10.8; 0.1‐32.0)
Country or region of origin, n (%)	
Mexico	13 (50)
Central America^b^	4 (14)
South America^c^	7 (27)
Other^d^	2 (8)
Preferred language^e^, n (%)	
Only Spanish	8 (31)
More Spanish than English	11 (42)
Both Spanish and English	7 (27)
Monthly income (US $), n (%)	
0-1000	11 (42)
1000-4999	15 (58)
Education, n (%)	
High school or less	16 (62)
Technical degree	5 (19)
College or postgraduate	5 (19)
Marital status^f^, n (%)	
Single	26 (100)
Has health insurance, n (%)	
Yes	15 (58)
HIV-related behaviors	
Ever tested for HIV, n (%)	
Yes	24 (92)
Tested for HIV in past 6 months, n (%)	
Yes	23 (89)
Current PrEP^g^ use, n (%)	
Yes	15 (58)
Number of sex partners in past 6 months, n (%)	
0	2 (8)
1^a^	3 (12)
2‐10	18 (69)
>10	3 (12)

a One participant from the in-depth interviews also participated in the focus group.

b Nicaragua, Honduras, El Salvador, and Guatemala.

c Colombia and Venezuela.

d Russia and the Philippines.

e No participants reported preferring only English or more English than Spanish.

f No participants reported being married.

g PrEP: pre-exposure prophylaxis.

#### In-Depth Interview Themes

In-depth interviews revealed four key themes and messages to guide the campaign’s development. The first theme focused on emphasizing prevention and awareness. Participants explained that it was important to promote PrEP availability, regular HIV testing, and diverse prevention strategies, including condom use. For example, one recommended that the content “include information about care and prevention, for example, advising them to use condoms if they have sexual relations, to take the PrEP pill, which is supposedly also used a lot here…” (34 years old, from El Salvador). Clear messaging around these measures was deemed essential for increasing awareness and encouraging proactive health behaviors.

The second theme was the need to highlight the accessibility of HIV services. Participants stressed that messages should convey that HIV services are accessible regardless of immigration status or insurance coverage. Specifically, participants proposed messages to encourage people to get tested such as, “I invite you to get tested for HIV and sexually transmitted diseases, the tests are free” (47 years old, from Mexico) and “We are all immigrants, we are all sisters” (44 years old, from Colombia). Emphasizing the availability of free services was viewed as critical to ensure that individuals do not feel excluded from care.

The third theme was the availability of culturally tailored services. Culturally tailored services, especially those provided by Latinos for Latinos, were identified as key to building trust and increasing engagement within the community. Among all, one participant explained why they sought services at a Latino-serving organization: “…first of all, they speak my language… Spanish, and they’re Latinos, so I felt more comfortable there” (44 years old, from Colombia). Culturally sensitive approaches were seen as essential for improving health care uptake.

The fourth theme was focused on confidentiality and safety. Ensuring confidentiality and safety for individuals accessing services was a top priority. Participants underscored that addressing privacy concerns is necessary to reduce fear of stigma or discrimination and to encourage HIV testing and HIV prevention. For example, a participant highlighted that, “to announce the availability of tests through social media and also tell them that it’s a test, one that isn’t shared with anyone, that’s confidential, so that they can also feel safe, knowing they won’t be judged or criticized” (32 years old, from Mexico).

In addition to these core themes, participants highlighted the need to address misinformation by dispelling myths and providing accurate information. Participants explained that messaging should be empowering and encourage individuals to take control of their health through knowledge, self-love, and personal responsibility. Real-life testimonials were seen as particularly powerful in reducing fear associated with HIV diagnoses and demonstrating that living with HIV is manageable, especially because participants felt more identified with individuals who had lived similar experiences, unlike celebrities or spokespeople who had not personally navigated these challenges. Participants underscored that reducing stigma remains crucial, particularly in challenging misconceptions linking HIV testing or positive status to promiscuity.

#### Preferred Platforms and Formats

Participants identified Facebook as a widely used platform for connecting with family, engaging with community content, and sharing information via videos, images, and links. Instagram was also valued for its visual content and broad reach.

Regarding content format, participants favored short, engaging videos over static images or text posts. Testimonial videos featuring relatable individuals were noted as especially impactful. Participants highlighted that there should be a diversity of personal experiences with HIV prevention and treatment. Visually appealing content such as colorful graphics, infographics, and engaging images was considered effective in capturing attention and conveying information succinctly.

Participants also emphasized the importance of using a natural, conversational tone to maintain credibility and trust. Overly polished or commercialized content was viewed as less authentic. A respectful approach, free of explicit or offensive material, was strongly recommended. A varied mix of testimonial videos, images, and infographics was suggested to keep content dynamic and engaging for the target audience.

#### Focus Group Recommendations

The results from the in-depth interviews were shared with a creative consulting agency, which developed 6 preliminary concept proposals for the social media campaign (Table 2). Focus group participants (n=7) then provided detailed feedback to further refine and culturally tailor these concepts to ensure relevance and resonance with the target population.

**Table 2.** Recommendations for the proposed social media concepts from the focus group with transgender Latina women conducted in June 2024 in the state of Washington.

Concept	Agency concept	Focus group recommendations
“For Your Family or Do it for Mom”	Framing HIV testing as an act of love for one’s family, centered on mothers	Broaden “family” to include friends, chosen family, and pets to reflect diverse support networks Acknowledge that not all mothers are supportive; offer alternative sources of support Incorporate emotional messaging emphasizing care and responsibility. Suggested messages: “Hazlo por tu familia (Do it for family),” “Creando historias (Creating stories),” “Hazte la prueba de VIH (Get tested for HIV),” and “Cuéntaselo a quien más confianza le tengas (Tell it to the person you trust most)” Include real photos and stories of transgender women for authenticity
“Land of the Free”	Aspirational imagery celebrating freedom and self-expression	Use ethereal, vaporous imagery reflecting exotic beauty and individuality Reference ’Todos me miran’ as cultural anchor of self-acceptance Suggested messages: “No hay excusas para cuidarte (There are no excuses not to take care of yourself),” “No hay límites para estar bien (There are no limits to being well),” and “Una existe (She exists)” Prioritize videos with some static images
“Living with HIV can be Fabulous”	Empowerment of individuals living with HIV	Portray empowered individuals living with HIV, emphasizing resilience Highlight theme: “Sin miedo a nada (Fearless)” Include real-life testimonials demonstrating positive living Use videos for dynamic storytelling
“Empowered and Protected”	Strength, protection, and unity within the community	Feature group activities (eg, dancing and community events) that convey unity and joy Avoid images reinforcing negative stereotypes Emphasize community solidarity and mutual support
“Celebrating Sexy, Healthy You”	Self-love is sexy and includes sexual health, enjoy your sexuality with confidence. Protect yourself from HIV	Depict a scene with someone getting ready for a romantic date, including condoms and PrEP^a^. Use voice-over to narrate internal thoughts (eg, “Siempre lista, siempre protegida [Always ready, always protected]”) Normalize HIV prevention as part of self-care Highlight relatable, everyday experiences Use video with personal narrative
“She Can Do It, So Can I”	Inspiration through role models within the community	Feature well-known community figures (eg, Wendy Guevara, Bambi Salcedo, and Alejandra Vogue) Highlight success stories overcoming adversity Reinforce empowerment and community leadership

a PrEP: pre-exposure prophylaxis.

Key recommendations are described in Table 2 and include broadening definitions of family, incorporating real-life testimonials, leveraging well-known cultural references and role models, and emphasizing empowerment, preparedness, and community support. These insights directly informed the campaign design.

The enthusiasm expressed by focus group participants for the social media campaign led to direct community involvement in campaign development. A total of 6 focus group participants joined the project team to collaborate on content creation, contributing to message refinement, imagery, and narrative development. This collaborative process resulted in the development of 4 final videos used in the social media campaign (MultimediaAppendices 1^4^).

### Phase 2: Online Surveys

Table 3 presents the sociodemographic characteristics and HIV prevention and care behaviors of the transgender Latina women who completed the online survey (n=100). No missing data were identified across the sociodemographic or HIV-related behavioral measures. Among participants, 54 completed the survey in Spanish and 46 completed the survey in English. The average age of respondents was 29.7 (SD 5.2) years. The majority (97/100, 97%) reported being born in the United States and having health insurance (98/100, 98%). Most (86/100, 86%) had completed some college or higher.

**Table 3.** Sociodemographic characteristics and HIV prevention behaviors of transgender Latina women survey respondents exposed to the social media campaign between November 2024 and December 2024 (n=100).

Variables	Value	95% CI
Sociodemographic characteristics		
Age (y), mean (SD)	29.7 (5.2)	—^a^
Race, n (%)		
Black	58 (58)	47.7‐67.8
Native Hawaiian or other Pacific Islander	2 (2)	0.3‐70
White	28 (28)	19.4‐38.2
Other	12 (12)	6.6‐20.3
Born in the United States, n (%)		
Yes	97 (97)	91.5‐99.0
Highest level of education, n (%)		
High school	6 (6)	2.8‐12.5
Technical or vocational training	8 (8)	4.1‐15.0
Some college or bachelor’s degree	69 (69)	59.2‐77.4
Graduate degree	17 (17)	11.0‐25.5
Annual household income (US $), n (%)		
10,000-19,000	2 (2)	0.3‐7.0
20,000-29,000	1 (1)	0.0‐5.4
30,000-39,000	6 (6)	2.8‐12.5
40,000-49,000	10 (10)	5.6‐17.2
50,000-74,000	51 (51)	40.9‐61.0
75,000-99,000	29 (29)	20.6‐39.1
>100,000	1 (1)	0.0‐5.4
Has medical insurance, n (%)		
Yes	98 (98)	93.0‐99.7
HIV-related behaviors		
Ever tested for HIV, n (%)		
Yes	97 (97)	91.5‐99.0
Self-reported HIV status, n (%)		
Positive	3 (3)	1.0‐8.5
Current PrEP^b^ use, n (%)		
Yes	62 (62)	52.2‐70.9
Alcohol use in past 12 months^c^, n (%)		
Never	41 (41.4)	31.8‐51.6
>once a month	2 (2.0)	0.3‐7.0
2‐4 times a month	21 (21.2)	14.4‐30.1
2‐3 times a week	30 (30.3)	22.0‐40.3
>4 times a week	5 (5.1)	2.0‐11.3
Used alcohol or drugs before last sexual encounter, n (%)		
None	51 (51)	40.9‐61.0
Alcohol	33 (33)	24.4‐43.0
Drugs	2 (2)	0.3‐7.0
Both	14 (14)	8.5‐22.1
Sex without a condom in past 6 months, n (%)		
Yes	69 (69)	59.2‐77.4
Number of sex partners in past 6 months, mean (SD; range)	2.5 (1.4; 0-8)	—
Group sex or orgies in past 12 months, n (%)		
Yes	33 (33)	24.4‐43.0

a Not applicable.

b PrEP: pre-exposure prophylaxis.

c One participant reported “Don’t Know”

Almost all respondents 97% (97/100; 95% CI 91.5‐99.0) had previously tested for HIV, with 44% (44/100; 95% CI 34.3‐53.7) indicating that they had tested within the last 6 months. Among all, 3 respondents reported being HIV positive, among which all reported currently taking ART. Approximately 58% (58, /100; 95% CI 48.3-67.7) reported alcohol use, with 35% (35/100; 95% CI 25.6‐44.4) indicating drinking alcohol 2 or more times a week. About 49% (49/100; 95% CI 39.2‐58.8) indicated using alcohol and/or drugs prior to their last sexual encounter, and 69% (69/100; 95% CI 59.2‐77.4) reported having sex without a condom in the last 6 months. Respondents had an average of 2.5 (SD 1.4) sexual partners in the past 6 months and approximately 33% (33/100; 95% CI 24.4‐43.0) reported engaging in group sex or orgies in the last year. Among respondents, 62% (62/100; 95% CI 52.2‐70.9) reported currently using PrEP.

When asked about the relevance of the social media content to their community, 98% (98/100; 95% CI 95.3‐100) indicated that the campaign was relevant. Qualitative responses to this question included, “I felt it was relevant as it is bringing out the happiness in trans women,” “It depicted a happy trans woman and I loved it,” “It reflects what I identify with,” and “It is an eye-opening [advertisement] to the general public that trans women are living happy lives.” Spanish responses (translated to English) included, “It gives hope to those infected and urges the community not to abandon them,” “It reassures those infected that we are all in this together,” and “This helps raise awareness in the community about HIV and its prevention measures, as well as not isolating those affected.”

When asked whether the campaign motivated them to take action such as seeking more information, talking to others, or changing behaviors, 91% (91/100; 95% CI 85.4‐96.6) indicated that it did. Qualitative responses explained that the campaign led some to engage in conversations with others about the topic, share the campaign with others, and seek more information about HIV prevention.

Beyond the survey data with the 100 respondents, performance data from Facebook and Instagram indicated that the 4 videos collectively had 211,355 views, a reach of 50,444 unique individuals, and 3267 link clicks over the course of the campaign period.

## Discussion

### Primary Findings

This study aimed to develop a social media campaign for transgender Latina women to increase awareness of HIV prevention and care services at a CBO. The use of a 2-phase, community-engaged approach allowed for the integration of participant perspectives throughout campaign development, ensuring that the content was both culturally relevant and responsive to the needs of the target population. The piloting of the campaign demonstrated its preliminary feasibility and acceptability of a culturally tailored social media content to reach transgender Latina women for HIV prevention services.

Findings from phase 1 revealed important themes related to prevention, accessibility, culturally tailored care, and confidentiality, which informed the development of the campaign content. Participants emphasized the importance of addressing stigma, misinformation, and the need for empowering messages delivered through accessible platforms such as Facebook and Instagram. Our findings align with prior research on HIV intervention development among transgender Latina populations, which emphasizes the importance of creating culturally responsive content that recognizes the diversity within the Latino community [33].

Notably, research focused on improving engagement in HIV prevention and care among transgender persons in the United States was limited to the last 2 decades but has expanded considerably in recent years [13]. In a recent systematic review of effective strategies to enhance engagement in HIV prevention among transgender populations, common features of successful interventions included community engagement in intervention design, testing with the target population to ensure acceptability, and addressing social determinants of health through empowerment-based and gender-affirming approaches [13]. Consistent with these findings, our study prioritized the unique needs of transgender Latina women and emphasized collaboration with community partners as well as pilot testing through survey assessments. The collaborative development process, which directly involved community members, strengthened the campaign’s cultural relevance and resonance.

Findings from phase 2 online surveys demonstrate the feasibility of our campaign in reaching transgender Latina women who may need HIV prevention and care services given high levels of reported engagement in HIV-related risk behaviors (eg, drug and alcohol use and sexual behaviors). Findings from phase 2 online surveys also suggest that the survey respondents reached through the online campaign may differ in important ways from those who participated in phase 1. Compared to the in-depth interview and focus group participants, phase 2 respondents were generally younger, predominantly US-born, had higher levels of education, and nearly all had health insurance. In addition, the use of ART and PrEP was relatively high.

These differences may reflect variation in social media platform use, online survey accessibility, or differing levels of engagement with HIV prevention services across subgroups of transgender Latina women. For instance, factors including digital access, health literacy, and language preferences play key roles in how social media messages reach communities and how they may be received [34]. A previous study that examined approaches to adapt mobile technology-based interventions for transgender Latina women found that the costs associated with smartphone ownership and internet use may limit engagement among socioeconomically disadvantaged groups [33]. Although the accessibility of mobile technology has improved over time, the diversity among phase 1 and phase 2 participants highlights the importance of considering heterogeneity within the broader population when designing and evaluating online versus other types of outreach efforts.

Transgender Latina women who are foreign-born may also face distinct barriers to HIV prevention and care compared to those who are US-born, as may those without health insurance [35]. These characteristics represent important social determinants of health that shape the contexts and experiences that influence HIV-related behaviors [36,37]. Although our study did not formally test for differences between phase 1 and phase 2 participants given the distinct purposes of each phase, the observed diversity between the 2 groups highlights the need to tailor outreach strategies to address varying levels of connectivity and access.

Despite these noted differences, most phase 2 participants rated the campaign as highly relevant to their community and reported being motivated to seek information, engage in conversations, and consider behavior change. Prior research identified strategies that successfully engaged transgender Latina women for an HIV prevention intervention trial, including leveraging existing social media platforms for outreach, ensuring the research team reflected the transgender Latina community, and relationship building with the community [38]. Similarly, our study reflects these effective approaches, while also highlighting a more organic, collaborative process in which ideas emerged during phase 1 in-depth interviews and informed the development of social media content. This iterative, community-engaged process proved critical for ensuring authenticity and resonance. Overall, our findings suggest that culturally grounded messaging has the potential to resonate across diverse segments of the population, while also underscoring the need for additional targeted efforts to reach subgroups who may be less connected to online platforms or who face more structural barriers to care.

### Limitations

Several limitations should be noted. First, survey respondents may not be representative of the broader population of transgender Latina women, particularly those who are foreign-born, uninsured, or face greater barriers to care. Furthermore, the survey was capped at the first 100 responses due to funding limitations; hence, respondents may not represent the characteristics of all who were reached by the campaign. The online recruitment strategy may have preferentially reached individuals who are more digitally connected and engage with social media. Second, all data were self-reported and may be subject to recall or social desirability bias, particularly in reporting HIV prevention behaviors. Third, the small sample size of the focus group limits generalizability, though the community-engaged approach helped ensure cultural appropriateness of the campaign materials. Finally, this study focused on short-term feasibility and acceptability; future research is needed to assess the longer-term impact of such campaigns on HIV prevention and care outcomes.

### Conclusion

This study demonstrates the potential of community-driven and culturally grounded social media strategies to enhance engagement in HIV prevention among transgender Latina women—a population often underrepresented in digital health initiatives. Our 2-phase, participatory study design centered on the voices, priorities, and lived experiences of transgender Latina women throughout campaign development. As participants who viewed the campaign and completed the survey rated the campaign as highly relevant to their community and reported being motivated to seek information, engage in conversations, and consider behavior change, our approach underscores the value of community partnership and cocreation in producing authentic and resonant health communication strategies.

Building on our promising results, future plans include expanding the campaign’s reach through strategic partnerships with CBOs and evaluating its impact on engagement in HIV-related services over time. In doing so, this work extends beyond a single campaign to inform how community-centered digital health strategies can be scaled. More broadly, our findings highlight the broader need for inclusive and participatory models of digital public health that address both structural barriers and the importance of cultural relevance. As social media and digital technologies continue to shape access to health and HIV-related information, integrating culturally tailored content that is co-created with the communities most affected may promote sustained engagement and build trust across diverse populations.

## Supplementary material

10.2196/79606 Multimedia Appendix 1 Social media post 1.

10.2196/79606 Multimedia Appendix 2 Social media post 2.

10.2196/79606 Multimedia Appendix 3 Social media post 3.

10.2196/79606 Multimedia Appendix 4 Social media post 4.

## Abbreviations

ART: antiretroviral therapy
CBO: community-based organization
LGBTQ+: lesbian, gay, bisexual, transgender/transsexual, queer, and other minority sexual orientations and gender identities
PrEP: pre-exposure prophylaxis
REDcap: Research Electronic Data Capture
UTB: Unified Theory of Behavior

## References

[1] Kanny D, Lee K, Olansky E (2024). Overview and methodology of the national HIV behavioral surveillance among transgender women—seven urban areas, United States, 2019-2020. MMWR Suppl.

[2] (2021). HIV infection, risk, prevention, and testing behaviors among transgender women—national HIV behavioral surveillance, 7 U.S. cities, 2019–2020. https://stacks.cdc.gov/view/cdc/105223.

[3] Yockey RA (2020). Hispanic transgender individuals: a forgotten diaspora. Hisp Health Care Int.

[4] Lett E, Asabor EN, Beltrán S, Dowshen N (2021). Characterizing health inequities for the US transgender Hispanic population using the behavioral risk factor surveillance system. Transgend Health.

[5] Bazargan M, Galvan F (2012). Perceived discrimination and depression among low-income Latina male-to-female transgender women. BMC Public Health.

[6] Bradford J, Reisner SL, Honnold JA, Xavier J (2013). Experiences of transgender-related discrimination and implications for health: results from the Virginia Transgender Health Initiative Study. Am J Public Health.

[7] Andrinopoulos K, Hembling J, Guardado ME, de Maria Hernández F, Nieto AI, Melendez G (2015). Evidence of the negative effect of sexual minority stigma on HIV testing among MSM and transgender women in San Salvador, El Salvador. AIDS Behav.

[8] Rich AJ, Williams J, Malik M (2020). Biopsychosocial mechanisms linking gender minority stress to HIV comorbidities among Black and Latina transgender women (LITE Plus): protocol for a mixed methods longitudinal study. JMIR Res Protoc.

[9] Nunes DDA, Pedreira GC, Katz-Wise SL Intersectionality and family support of Latin American transgender individuals: a scoping review. Int J Transgend Health.

[10] Hajek A, König HH, Blessmann M, Grupp K (2023). Loneliness and social isolation among transgender and gender diverse people. Healthcare (Basel).

[11] Furuya A, Ransome Y, Kawachi I (2025). Community connectedness as a source of adherence to HIV prevention behaviors and resilience among transgender women of color in New York City, 2020-2022. Am J Public Health.

[12] Cooney EE, Reisner SL, Saleem HT (2022). Prevention-effective adherence trajectories among transgender women indicated for PrEP in the United States: a prospective cohort study. Ann Epidemiol.

[13] Crepaz N, Peters O, Higa DH, Mullins MM, Collins CB (2025). Identifying effective strategies for improving engagement in HIV prevention and care among transgender persons in the United States: a systematic review. AIDS Behav.

[14] Lee K, Trujillo L, Olansky E (2022). Factors associated with use of HIV prevention and health care among transgender women—seven urban areas, 2019-2020. MMWR Morb Mortal Wkly Rep.

[15] Smart BD, Alonzo J, Mann-Jackson L (2024). Transgender Latinas’ perspectives on HIV PrEP uptake, condom use, and medically supervised gender-affirming hormone therapy: insights from CHICAS qualitative interviews. AIDS Educ Prev.

[16] Aguayo-Romero RA, Valera G, Cooney EE, Wirtz AL, Reisner SL (2025). “When Somebody Comes into This Country and You Are Trans on Top of That Is Like You Got… Two Strikes on You”: intersectional barriers to PrEP use among Latina transgender women in the Eastern and Southern United States. Int J Environ Res Public Health.

[17] Li DH, Brown CH, Gallo C (2019). Design considerations for implementing eHealth behavioral interventions for HIV prevention in evolving sociotechnical landscapes. Curr HIV/AIDS Rep.

[18] Schnall R, Travers J, Rojas M, Carballo-Diéguez A (2014). eHealth interventions for HIV prevention in high-risk men who have sex with men: a systematic review. J Med Internet Res.

[19] Taggart T, Grewe ME, Conserve DF, Gliwa C, Roman Isler M (2015). Social media and HIV: a systematic review of uses of social media in HIV communication. J Med Internet Res.

[20] Nguyen LH, Tran BX, Rocha LEC (2019). A systematic review of eHealth interventions addressing HIV/STI prevention among men who have sex with men. AIDS Behav.

[21] Lee JJ, Aguirre Herrera J, Cardona J (2022). Culturally tailored social media content to reach Latinx immigrant sexual minority men for HIV prevention: web-based feasibility study. JMIR Form Res.

[22] Padgett DK (2012). Qualitative and Mixed Methods in Public Health.

[23] Malterud K, Siersma VD, Guassora AD (2016). Sample size in qualitative interview studies: guided by information power. Qual Health Res.

[24] Hampton B, Brinberg D, Peter P, Corus C (2009). Integrating the unified theory and stages of change to create targeted health messages. J Applied Social Pyschol.

[25] Lindsey MA, Chambers K, Pohle C, Beall P, Lucksted A (2013). Understanding the behavioral determinants of mental health service use by urban, under-resourced Black youth: adolescent and caregiver perspectives. J Child Fam Stud.

[26] Tolley EE, Ulin PR, Mack N, Robinson ET, Succop SM (2016). Qualitative Methods in Public Health: A Field Guide for Applied Research.

[27] Bandura A (1982). Self-efficacy mechanism in human agency. Am Psychol.

[28] Bandura A, Adams NE (1977). Analysis of self-efficacy theory of behavioral change. Cogn Ther Res.

[29] St George SM, Harkness AR, Rodriguez-Diaz CE, Weinstein ER, Pavia V, Hamilton AB (2023). Applying rapid qualitative analysis for health equity: lessons learned using “EARS” with Latino communities. Int J Qual Methods.

[30] Lewinski AA, Crowley MJ, Miller C (2021). Applied rapid qualitative analysis to develop a contextually appropriate intervention and increase the likelihood of uptake. Med Care.

[31] Wallerstein N, Duran B, Oetzel JG, Minkler M (2018). Community-Based Participatory Research for Health: Advancing Social and Health Equity.

[32] Harris PA, Taylor R, Thielke R, Payne J, Gonzalez N, Conde JG (2009). Research electronic data capture (REDCap)—a metadata-driven methodology and workflow process for providing translational research informatics support. J Biomed Inform.

[33] MacCarthy S, Barreras JL, Mendoza-Graf A, Galvan F, Linnemayr S (2019). Strategies for improving mobile technology-based HIV prevention interventions with Latino men who have sex with men and Latina transgender women. AIDS Educ Prev.

[34] Escobar-Viera CG, Melcher EM, Miller RS (2021). A systematic review of the engagement with social media-delivered interventions for improving health outcomes among sexual and gender minorities. Internet Interv.

[35] Lee JJ, Leyva Vera CA, Ramirez J (2023). “They already hate us for being immigrants and now for being trans-we have double the fight”: a qualitative study of barriers to health access among transgender Latinx immigrants in the United States. J Gay Lesbian Ment Health.

[36] Lee JJ, Yu G (2019). HIV testing, risk behaviors, and fear: a comparison of documented and undocumented Latino immigrants. AIDS Behav.

[37] Sheehan DM, Trepka MJ, Dillon FR (2015). Latinos in the United States on the HIV/AIDS care continuum by birth country/region: a systematic review of the literature. Int J STD AIDS.

[38] Alonzo J, Mann-Jackson L, Tanner AE (2025). Increasing engagement of Spanish-speaking transgender Latinas in research: strategies from ChiCAS, a community-based participatory research intervention trial. Health Educ Res.

